# The Quorum Sensing System of *Yersinia enterocolitica* 8081 Regulates Swimming Motility, Host Cell Attachment, and Virulence Plasmid Maintenance

**DOI:** 10.3390/genes9060307

**Published:** 2018-06-20

**Authors:** Yen-Kuan Ng, Marco Grasso, Victoria Wright, Vanina Garcia, Paul Williams, Steve Atkinson

**Affiliations:** 1 Centre for Biomolecular Sciences, School of Life Sciences, University of Nottingham, Nottingham NG7 2RD, UK; ngyenkuan2@yahoo.co.uk (Y.-K.N); m.grasso83@gmail.com (M.G.); Vanina.Garcia@nottingham.ac.uk (V.G.); Paul.Williams@nottingham.ac.uk (P.W.); 2 Deep Seq, Centre for Genetics and Genomics, School of Life Sciences, University of Nottingham, Nottingham NG7 2UH, UK; Victoria.Wright@nottingham.ac.uk

**Keywords:** *Yersinia enterocolitica*, quorum sensing, LuxR, *N*-acylhomoserine lactones, motility, cell attachment, virulence plasmid maintenance

## Abstract

Although *Yersinia enterocolitica* genomes are highly heterogeneous, they contain a conserved *N*-acylhomoserine lactone-dependent (AHL) quorum sensing (QS) system consisting of the *luxR* and *luxI* orthologs *yenR* and *yenI* respectively. Certain hypervirulent strains also contain a putative orphan *luxR* gene, *ycoR*, that is not linked to an AHL synthase. To explore the contribution of *yenR/yenI/ycoR* to QS-dependent phenotypes in *Yersinia enterocolitica* strain 8081, single and multiple mutants were constructed. AHL profiling identified *N*-(3-oxohexanoyl) homoserine lactone, *N*-hexanoylhomoserine lactone, and *N*-(3-oxoseptanoyl) homoserine lactone as the most abundant. The AHL profiles of the *yenR*, *ycoR* and *yenR/ycoR* mutants were similar to the parent suggesting that the two LuxR homologues do not regulate AHL production while the *yenI* mutants were AHL-negative. A role for QS in swimming motility and cell attachment was demonstrated. Down-regulation of the virulence plasmid partition gene, *spyA*, in *yenI* and *yenI*/*yenR*/*ycoR* mutants is consistent with the greater loss of the *Y. enterocolitica* pYVe virulence plasmid in the *yenI* mutant during serial passage at 37 °C but not at 22 °C. A role for QS-regulated *spyA* in virulence plasmid maintenance is suggested.

## 1. Introduction

Bacterial populations respond to external stimuli by producing and transducing chemical signal molecules in a cell density-dependent process known as quorum sensing (QS). QS systems have been described in a variety of plant and animal pathogens, including Gram-negative and Gram-positive bacteria, in which *N*-acylhomoserine lactones (AHLs), 2-alkyl-4-quinolones, and small peptides, for example, form the diffusible signal molecule component (for reviews see [1,2]). In Gram-negative bacteria, the AHL synthase genes (coded for by e.g., orthologs of *Vibrio fischeri luxI*) are responsible for signal molecule synthesis, while AHL signal transduction is usually carried out by a regulator belonging to the LuxR family of proteins.

The three *Yersinia* species that are pathogenic for humans are psychrotrophic and grow well between 4 °C and 42 °C and, as such, are well adapted to survive outside their mammalian hosts. Upon infection they adapt rapidly to a temperature shift to 37 °C where they cause differing diseases ranging from bubonic, pneumonic and septicaemic plague (*Yersinia pestis*) to gastroenteritis (*Yersinia pseudotuberculosis* and *Yersinia enterocolitica*). The virulence of the pathogenic *Yersinia* depends on the presence of an ~70-kb virulence plasmid, termed pYve, in *Y. enterocolitica* that encodes the *Yersinia* outer protein (Yop) virulon. This consists of a type III secretion system (T3SS) which facilitates the injection of multiple Yop effector proteins directly into the cytosols of eukaryotic cells to subvert host cell signalling pathways. T3SS is tightly regulated by environmental conditions and, in particular, the Ca^2+^ ion concentration and temperature which ensures that Yops are normally secreted at 37 °C but not at 22 °C (for reviews see [3,4,5]).

*Y. pseudotuberculosis* and *Y. pestis* possess two pairs of convergently transcribed *luxI/R* orthologs (*ypsI*/*R*, *ytbI*/*R* and *ypeI*/*R* and *yepI*/*R*, respectively). Both species produce the same four major AHLs, namely *N*-(3-oxohexanoyl) homoserine lactone (3-oxo-C6-HSL), *N*-hexanoylhomoserine lactone (C6-HSL), *N*-octanoylhomoserine lactone (C8-HSL), and *N*-(3-oxooctananoyl) homoserine lactone (3-oxo-C8-HSL) [6,7,8]).

In *Y. pseudotuberculosis*, QS controls virulence-related phenotypes including biofilm production on the cuticle of the nematode worm, *Caenorhabditis elegans*, the T3SS system [9], flagella-mediated motility and cell aggregation [10]. In comparison, few phenotypes have been associated with the single pair of convergently transcribed (Figure S1) *luxR*/*I* orthologs (*yenI/R*) in *Y. enterocolitica*. In *Y. enterocolitica* strain 90/54 *yenI* mutants, swimming motility is temporally delayed in plate assays and swarming motility is abolished [11], while the equivalent *yenI* mutant in *Y. enterocolitica* strain 10460 is unaffected in either swimming or swarming. In the 90/54 *yenI* mutant, swimming can be restored by genetic complementation with a plasmid-borne copy of *yenI* but not by the provision of exogenous AHLs [11]. Tsai and Winans [12] showed that for *Y. enterocolitica* JB580 [13], a restriction endonuclease and methyltransferase variant of strain 8081, YenR acts as an apoprotein. In the absence of AHLs, YenR activates the expression of a regulatory RNA *yenS*, which is proposed to bind to the YenI mRNA, blocking translation. When bound to AHLs, *YenR* cannot activate *yenS* expression, and this leads to an increase in *yenI* expression. In this system, swarming but not swimming motility increases in the *yenI* mutant and can be restored to the parental non-swarming phenotype with exogenous addition of 3-oxo-C6-HSL. Given that three different *Y. enterocolitica* strains all show different QS-dependent and QS-independent motility phenotypes illustrates the existence of regulatory diversity within the same QS network. Since *Y. pseudotuberculosis ypsI/R* negatively regulates motility while *ytbI/R* activates motility (swarming remains unaffected), we previously suggested that the control of motility in these two members of the *Yersiniae* was mechanistically distinct because of the lack of a second AHL-dependent QS system in *Y. enterocolitica* [11].

In the present study, we sought to identify the QS-controlled phenotypes in *Y. enterocolitica* 8081 and establish a role for gene YE1026 which that is annotated as a potential QS regulator in the published genome sequence [14]. A sequence analysis of YE1026 suggests that it is a *luxR* ortholog that we now term *ycoR.* To determine whether *ycoR* contributes to QS, *yenI*, *yenR* and *ycoR* were each mutated either individually or in combination, and their AHL profiles were examined. From these data, we show that the *yenI*/*yenR*/*ycoR* QS system in *Y. enterocolitica* strain 8081 contributes to motility, eukaryotic cell attachment, and maintenance of the type III secretion system (TTSS) virulence plasmid, pYVe.

## 2. Materials and Methods

### 2.1. Strains and Growth Conditions

The strains and plasmids used in this study are listed in Tables S1 and S2 respectively. Unless otherwise stated, all strains were grown statically or with shaking at 200 rpm in L broth Lennox (YLB) or on L broth Lennox agar plates [15] at either 22 °C or 37 °C. Where necessary, growth media were buffered to pH 6.8 with (3-*N*-morpholino) propanesulphonic acid (YLB_mops_) to reduce the alkaline hydrolysis of AHLs during bacterial growth [16]. The following supplements were used at the indicated final concentrations: chloramphenicol, 30 μg mL^−1^; kanamycin, 50 μg mL^−1^; streptomycin, 50 μg mL^−1^; ampicillin, 50 μg mL^−1^; nalidixic acid, 15 μg mL^−1^; isopropyl-β-d-thiogalactopyranoside (IPTG), 40 μg mL^−1^; and 5-Bromo-4-chloro-3-indoyl-β-d-galactopyranoside (Xgal), 40 μg mL^−1^.

### 2.2. Construction of the Quorum Sensing Mutants

The QS mutants were constructed using the modified λ red recombinase method of Datsenko and Wanner [17] adapted for *Y. enterocolitica* [18]. This PCR-based method allows the in vivo recombination of a deletion insertion mutation into a gene of interest using the helper plasmid, pAJD434, which encodes phage λ red recombinase. pAJD434 was electroporated into *Y. enterocolitica* 8081, and positive transformants were selected on trimethoprim^100^ agar plates. Primers (Table S3) that contained homologous sequences to *yenI*, *yenR* or *ycoR* were used to amplify kanamycin, chloramphenicol (Figure S1), and streptomycin cassettes from pUC4k, pACYC184, and pHP45Ω, respectively. Following amplification, the flanking region of each QS gene along with the appropriate antibiotic cassette were pooled in a second round of amplification following the method of Derbise et al. (2003) [19]. An additional enrichment step was introduced following this PCR step in which the product was used as a template using the two flanking primers. For each QS gene, the resulting PCR product was transformed into *Y. enterocolitica* 8081 pAJD434, and potential mutants were selected with appropriate antibiotics, cured of pAJD434 at 37 °C, and three independent mutants were confirmed by PCR, Southern hybridization, and sequencing (data not shown). Double and triple mutants were constructed using the same method as the appropriate newly constructed single or double mutants.

To complement the QS mutations, primers designed to amplify *yenI*, *yenR* or *ycoR* (Table S3) were used with Expand High Fidelity polymerase (Sigma-Aldrich, Gillingham, Dorset, UK) and *Y. enterocolitica* 8081 chromosomal DNA as a template. PCR products were cloned into pGEMT-Easy (Promega, Southampton, Hants, UK), transformed into *Escherichia coli* JM109 and sequenced. Each gene was then sub-cloned into pHG327 as *Kpn*I/*Bam*HI or *Sal*I/*Pst*I fragments, transferred to the low copy number vector, pME3087, as *Kpn*I/*Bam*HI (*yenI* and *yenR*) or *Bam*HI/*Pst*I (*ycoR*) fragments, and transformed into *E. coli* S17-1 λ *pir*^+^ before being conjugated into *Y. enterocolitica* 8081.

### 2.3. N-Acylhomoserine Lactone Profiling

Comprehensive profiling of the AHLs produced (as relative molar ratios) by the *Y. enterocolitica* parent and QS mutants was performed as described before [6]. Cell-free supernatant extracts were taken from cultures grown optimally for AHL production at 30 °C in YLB_mops_, extracted twice with ethyl acetate and analysed using liquid chromatography coupled to hybrid quadrupole–linear ion trap QqQLIT mass spectrometry (LC-MS).

### 2.4. Quantitative Reverse Transcription PCR

RNA was isolated during the late-log phase (OD_600_ 0.75) following growth at 22 °C and 37 °C, temperatures to represent contrasting environmental and host conditions consistent with the biphasic lifestyle of *Y. enterocolitica*. All experiments were done in triplicate. To extract RNA, 4 mL of culture was added to 8 mL of RNAprotect^TM^ (Qiagen, Manchester, UK). Cells were pelleted, and RNA was extracted using RNeasy (Qiagen, Manchester, UK) midi columns with on-column DNaseI digestion as per the manufacturer’s instructions. RNA was quantified (NanoDrop ND-1000), and sample integrity was confirmed using the Agilent Bioanalyzer 2100 with 2100 Expert software (Agilent, Technologies, Stockport, Cheshire, UK). The total RNA samples were treated with DNAseI for 1 h, washed and eluted in 50 µL of HPLC grade water and stored at −20 °C until required. First strand cDNA synthesis was performed using a cDNA synthesis kit (GE Healthcare, Little Chalfont, Bucks, UK) according to the manufacturer’s instructions. The cDNA was purified with the MinElute PCR purification system (Qiagen, Manchester, UK). The *dnaE* gene was used as an endogenous control, and Primer Express software used to design primer pairs that were optimised for efficiency by varying the ratios. Quantitative reverse transcription PCR (qRT-PCR) was performed using the SYBR Green PCR Master Mix according to manufacturer’s protocol (Applied Biosystems, Foster City, California, USA). The comparative cycle threshold method (C_T_) was used to determine the changes in the steady state mRNA levels of the target gene in the parent compared with the QS mutants and expressed relative to the levels of the internal housekeeping control gene, *dnaE* [20].

### 2.5. Motility, Autoagglutination and Haemagglutination Assays

For liquid culture motility assays, the parent and QS mutants were grown in 1/10 YLB_mops_ and examined under a phase contrast microscope. Motility was assessed after growth for 9 h and after 24 h, as previously described [21].

A modified agglutination assay was performed [22] in which the growth of autoagglutination-positive (Agg^+^) strains resulted in an irregularly-edged layer of agglutinated bacteria, forming a flocculate covering the bottom of the tube.

Haemagglutination was performed using 50 μL of an overnight culture of the appropriate strain grown at 22 °C or 37 °C that was added to a V-well plate along with 50 μL of phosphate buffered saline (PBS) and two drops of a 1% (in PBS) sheep blood suspension. The plate was then incubated at 4 °C for 3 h or 24 h before examination.

### 2.6. Cell Attachment Assays

Attachment assays were performed using a modified method of Di Biase et al. [23]. Human colonic adenocarcinoma (Caco-2) cells were seeded in a 24-well culture plate to a final concentration of 1 × 10^5^ cells mL^−1^ and incubated at 37 °C, 5% CO_2_ for 48 h to allow for a semi-confluent monolayer. Bacteria were grown overnight in 5 mL YLB with antibiotics at 30 °C, sub-cultured for 2 h at 37 °C in Brain Heart Infusion (BHI) [24], diluted to a give a multiplicity of infection (MOI) of 100 CFU cell^−1^ in Dulbecco’s Modified Eagle’s Medium (DMEM) without antibiotics, added to the Caco-2 monolayer, and incubated for 3 h at 4 °C to allow attachment. Cells were washed (×5) with DMEM to remove unattached bacteria, lysed using cold 0.1% Triton X-100 in PBS, serial diluted, and spotted on YLB_mops_ plates for colony counting. Attachment was reported as the number of adherent bacteria/100 Caco-2 cells.

### 2.7. Virulence Plasmid Loss and spyA Expression

Congo Red (CR) can be used to detect the presence of the *Yersinia* spp. virulence plasmid [25]. On magnesium oxalate agar incorporating Congo red (CR-MOX), *Y. enterocolitica* colonies containing the pYVe plasmid are small and red, but they are large and white/pink if the plasmid is absent [26]. To quantify the loss of pYVe, bacteria were grown overnight in 10 mL YLB at 37 °C, and sub-cultured 1:1000 in fresh medium every day over a 10 day period. Each day, the culture was serially diluted, plated on CR-MOX plates, and incubated at 22 °C or 37 °C for 24 h. Colonies were counted and plotted as a percentage of white/pink (plasmid negative colonies).

To examine the expression of *spyA*, *lux*-based promoter fusions were constructed as previously described [27] with modifications. PCR primers were designed to amplify the promoter regions of *spyA* incorporating *Apa*I and *Not*I restriction sites. The resulting PCR products were cloned into similarly digested pBluescript to give pBlue::P*_spyA_*. The *luxCDABE* cassette was excised from pBlue*lux* [27] and cloned into pBlue::P*_spyA_* as a *Sac*I fragment to give pBlue::P*_spyA_*::*luxCDABE*, after which *P_spyA_*::*luxCDABE* was cloned as an *Apa*I/*Not*I fragment into similarly digested pDM4 [28] to give pYK801. The newly constructed *P_spyA_::luxCDABE* was conjugated into the parent *yenI*, *yenR*, *ycoR*, *yenR/ycoR* and *yenl/yenR/ycoR* mutants, and *spyA* expression was determined at 22 °C and 37 °C as a function of temperature and growth phase using a Tecan combined spectrophotometer/luminometer with expression calculated as relative light units (bioluminescence)/OD_405_ against time [27] and plotted as Area Under the Curve (AUC) for a 12 h period. Growth curves for all the strains harboring the *lux*-fusion were carried out to ensure that growth was not affected by the plasmid vector.

## 3. Results

### 3.1. Yersinia enterocolitica 8081 Possesses an Orphan luxR Ortholog

The full genome sequence of *Y. enterocolitica* 8081 [14] contains an open reading frame (ORF), annotated as YE1026. Its amino acid sequence is most similar to the LuxR orthologs SmaR (*Serratia* sp.), EchR (*Erwinia chrysanthemi*), ExpR (*Erwinia carotovora*) and EsaR (*Erwinia (Pantoea*) *stewarti*) (40–57%) and also has similarities to *Yersinia* LuxR orthologs, including YpsR, YtbR, YpeR, YenR and YukR (40–43%) (data not shown). Upon closer examination, the predicted protein motif analysis revealed that the *N*- and *C*-terminal domains of this 240 amino acid ORF possess key conserved residues that match the consensus for LuxR orthologs [29] and therefore, we term YE1026, YcoR. The *ycoR* gene is not genetically linked to a *luxI* ortholog suggesting it is an orphan or solo *luxR* [30] and is distantly located ~650 kb upstream of the *yenI/R* locus. Examination of the region upstream of *ycoR* does not reveal any sequence similarity to Yen Box I or II, the YenR binding domains described by Tsai and Winans [12], but divergent to *YcoR* and close to YE1027 is a putative transcriptional repressor, *sfsB* (YE1026A).

Howard et al. [31] reported that *Y. enterocolitica* genomes are highly heterogeneous and given that *Y. enterocolitica* 8081 possesses *ycoR*, we sought to investigate whether this heterogeneity extends to the *yenI/yenR/ycoR* QS system. Using DNA microarray data published by Howard et al., we examined 98 *Y. enterocolitica* strains isolated from humans, sheep, pigs and cattle in the UK and USA belonging to the pathogenic biogroup 1A, the non-pathogenic biogroup 1B and the weakly pathogenic biogroups 2, 3 and 4 for *yenI*, *yenR* and *ycoR*, respectively. Strains which showed the presence of *yenI/yenR* and *ycoR* in the divergent list (their presence was, therefore, ambiguous) were checked by PCR to confirm the presence of each gene. Ten percent of the strains, all isolated from human hosts in the USA and belonging to the pathogenic biogroup 1B, possessed the *ycoR* locus. None of the remaining strains isolated from the UK possessed *ycoR*.

**Figure 1:**
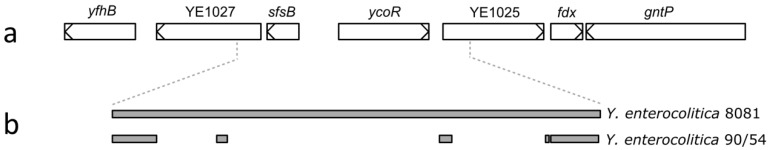
Sequence comparison of the *ycoR* locus in the *Yersinia enterocolitica* 8081 and 90/54 strains. In 8081, *ycoR* lies between YE1027 and YE1025 (**a**), but the region is absent in strain 90/54 (**b**) (areas of significant homology are shaded grey).

Because only the US strains possessed a second *luxR* ortholog, we examined, in more detail, the *ycoR* locus of *Y. enterocolitica* 8081 (a US strain, biogroup 1B) and compared the equivalent region in strain 90/54 (a UK biogroup 1B). The region was amplified using PCR, and the products were sequenced. A comparison of the two loci indicated the position at which *ycoR* is absent in the 90/54 strain (Figure 1). Further analysis revealed that only *ycoR* is absent at this locus, with YE1025, *sfsB* YE1027 and the intergenic region remaining intact in the 90/54 strain (Figure 1).

### 3.2. Comprehensive N-Acylhomoserine Lactone Profiling of the Yersinia enterocolitica 8081 Parent and Quorum Sensing Mutants

**Figure 2:**
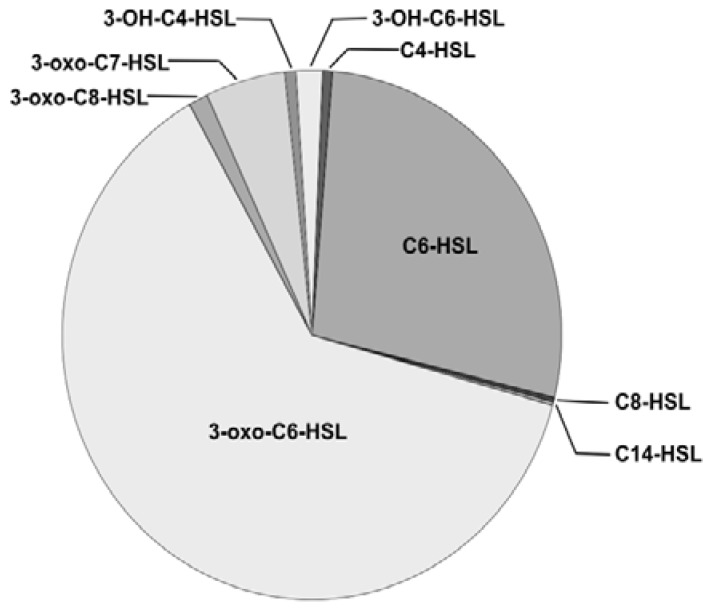
Pie chart illustrating the relative molar ratios of AHL lactone-dependent (AHLs) identified in *Y. enterocolitica* 8081. *N*-(3-oxohexanoyl)homoserine lactone (3-oxo-C6-HSL) (62.4%) is the major AHL, followed by *N*-hexanoylhomoserine lactone (C6-HSL (27.4%) and *N*-(3-oxoheptanoyl) homoserine lactone (3-oxo-C7-HSL) (5.1%). Low levels of *N*-(3-oxooctanoyl)homoserine lactone (3-oxo-C8-HSL); *N*-(3-hydroxybutanoyl)homoserine lactone (3-OH-C4-HSL); *N*-(3-hydroxyhexanoyl)homoserine lactone (3-OH-C6-HSL); *N*-butanoylhomoserine lactone (C4-HSL) ; *N*-octanoylhomoserine lactone (C8-HSL); (N-tetradecanoylhomoserine lactone (C14-HSL) were also present.

In frame deletions of *Y. enterocolitica* 8081, *yenI*, *yenR*, and *ycoR* were constructed using the λ red recombinase system [19]. The single mutants were used to construct *yenI/yenR*, *yenI/ycoR*, *yenR/ycoR* double and *yenI/yenR/ycoR* triple mutants, respectively. The AHL profile of the parent 8081 strain was obtained by LC-MS analysis of cell-free culture supernatants [6]. Sixteen AHLs were identified, 11 of which have not previously been documented for *Y. enterocolitica* (Table S4). Figure 2 illustrates the relative molar ratios of the most prevalent AHLs produced by *Y. enterocolitica* 8081. The major AHL was 3-oxo-C6-HSL (62.4%), followed by C6-HSL (27.4%) while the odd acyl chain AHL, *N*-(3-oxoheptanoyl) homoserine lactone (3-oxo-C7-HSL), represented 5.1%. A full list of the relative proportions of the AHLs produced is summarized in Table S4.

The AHL profiles of the QS mutants (*yenI*, *yenR*, *ycoR*, *yenI/yenR*, *yenI/ycoR*, *yenR/ycoR*, and *yenI/yenR/ycoR*) were also examined using LC-MS analysis. The *yenR*, *ycoR*, and *yenR/ycoR* mutants all produced similar AHL profiles to the parent, suggesting that the LuxR homologues play no part in determining the quantity or nature of the AHLs produced. AHL production in all mutants that included a *yenI* mutation (i.e., *yenI/yenR*, *yenI/ycoR* and *yenI/yenR/ycoR mutants*) was abolished (Figure S2).

### 3.3. Phenotypic Analysis Reveals a Role for Quorum Sensing in Swimming Motility and Eukaryotic Cell Attachment but Not Type Three Secretion.

For *Y. pseudotuberculosis*, we have previously shown that QS is involved in the modulation of components of the TTSS including Yop chaperones and their effectors [9] and we therefore examined whether similar regulatory networks to that of *Y. pseudotuberculosis* may exist in *Y. enterocolitica*. However, Yop secretion assays comparable to those performed for *Y. pseudotuberculosis* [9] did not support a role for QS in promoting Yop release at non-permissive temperatures (data not shown).

**Figure 3:**
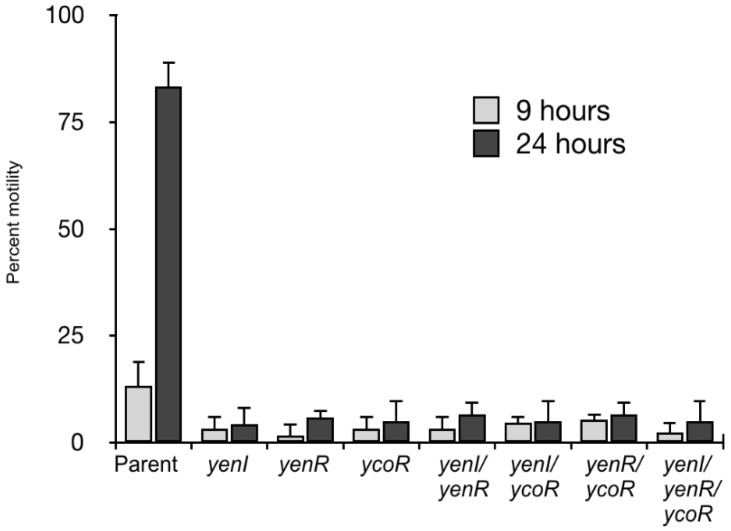
Swimming motility in liquid culture. After 9 h and 24 h, the parent population was substantially more motile than the quorum sensing (QS) mutants, with a particularly marked difference after 24 h.

As QS has previously been shown to regulate motility in the *Y. enterocolitica* strains 90/54 and JB580 [11,12], we examined swimming and swarming in the *Y. enterocolitica* 8081 parent and QS mutants grown on semi-solid swim and swarm agar plates, as previously described [11,12]. Surprisingly, despite the fact that the *Y. enterocolitica* strains 90/54, [11] and JB580 [12] have been reported to have QS-regulated swimming or swarming motility phenotypes, no obvious differences were observed between the *Y. enterocolitica* 8081 parent and QS mutants on semi-solid medium (data not shown). However, when the parent and QS mutants were grown in liquid culture and examined under a microscope, the percentage of the population which were motile was low at 9 h, but after 24 h, the percentage of motile QS mutants remained low, whereas over 80% of the parent population was swimming (Figure 3).

Expression of the pYVe virulence plasmid located autoagglutination gene, *yadA*, is induced at 37 °C by the temperature-dependent transcriptional activator VirF [32]. We have previously shown that the *Y. pseudotuberculosis* QS system is involved in regulating aggregation [10], and we therefore surmised that QS may play a part in YadA-mediated aggregation in *Y. enterocolitica*. To investigate this, we first performed qRT-PCR experiments which showed that *yadA* expression in the *yenR/ycoR* and *yenI/yenR/ycoR* mutants at 37 °C was reduced >3-fold (data not shown).

Agglutination and haemagglutination are considered to be indicators of virulence in *Y. enterocolitica* and are attributed to YadA [33]. To determine whether the *yadA* expression data is related to a cell attachment phenotype, we conducted agglutination and haemagglutination assays in the *Y. enterocolitica* 8081 parent and QS mutants using the modified assays described by Laird et al. [22]. All strains were agglutination-positive at 22 °C and 37 °C, and haemagglutination-negative at 22 °C, but haemagglutination-positive at 37 °C (data not shown).

**Figure 4:**
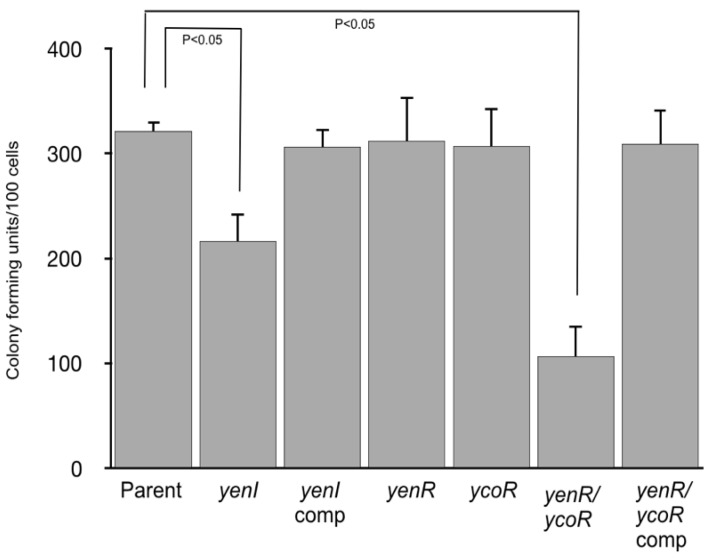
Attachment to human colorectal adenocarcinoma Caco-2 cells. The *yenI* and *yenR*/*ycoR* mutants showed a reduced ability to attach to Caco-2 cells. This phenotype was restored when functional copies of either *yenI* or *yenR* and *ycoR*, respectively, were supplied *in trans* (*yenI* comp and *yenR/ycoR* comp).

Although QS does not appear to influence these phenotypes, the ability of pathogenic *Y. enterocolitica* to adhere to and invade tissue culture cells is an important marker for virulence [34,35]. We therefore investigated whether *Y. enterocolitica* 8081 could invade or adhere to Caco-2 cells. Although there were no differences between the parent and the QS mutants in their ability to invade the Caco-2 cells (data not shown), Figure 4 shows that in attachment assays, the number of CFUs/100 Caco-2 cells was ~320 in the parent but reduced to ~210 and ~110 in the *yenI* and *yenR*/*ycoR* mutants, respectively.

It is notable that the *yenR* and *ycoR* single mutants showed levels of attachment to Caco-2 cells that were comparable with the parent. Attachment was restored to parental levels when functional copies of *yenI* or *yenR*/*ycoR* were respectively provided *in trans*.

### 3.4. Quorum Sensing and Virulence Plasmid Maintenance

During sub-culture experiments at 37 °C, which were intended to examine the growth rate of the QS mutants when compared to the parent, each strain was grown on CR-MOX plates to check for the presence of the virulence plasmid. While conducting these experiments, we noted that the *yenI* mutant appeared to rapidly lose the virulence plasmid, as indicated by an increase in the number of large, white, pYVe-negative colonies compared with their small, red, pYVe-positive counterparts [20]. These observations raised the intriguing possibility that QS is involved in the regulation of plasmid partition and prompted us to use qRT-PCR to examine the expression of the virulence plasmid partition gene *spyA* in the parent and QS mutants. The data obtained showed that expression was reduced in each of the *yenI* and *yenI*/*yenR*/*ycoR* mutants, respectively, by ~2–3-fold (data not shown).

**Figure 5:**
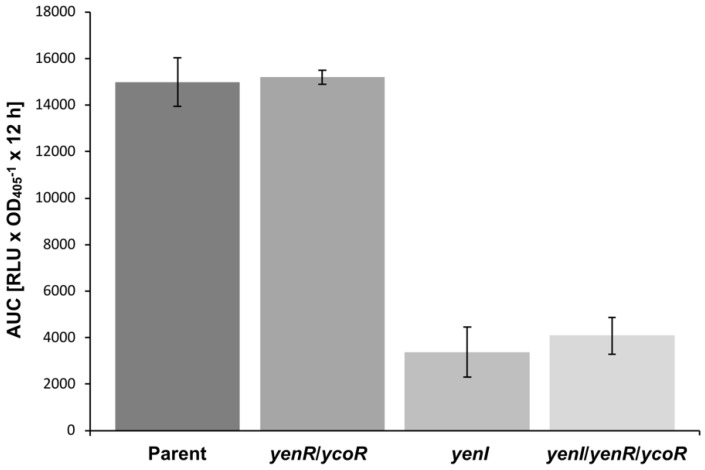
Expression of *spyA* over 12 h in the parent and QS mutants at 37 °C. The expression of the P*_spyA_*::*luxCDABE* promotor fusion was comparable for the parent and *yenR*/*ycoR* mutant but was reduced ~4-fold in the *yenI* and *yenI*/*yenR*/*ycoR* mutants.

To obtain further evidence of a role for QS in influencing *spyA* transcription, we constructed *lux*-based reporter fusions to the *spyA* promoter in three mutants and examined expression as a function of growth over 12 h in liquid culture. Figure 5 shows that *spyA* expression at 37 °C in the parent and *yenR*/*ycoR* mutant were similar and approximately 4-fold higher than *spyA* expression in the *yenI* and *yenI*/*yenR*/*ycoR* mutants, consistent with the qRT-PCR data.

**Figure 6:**
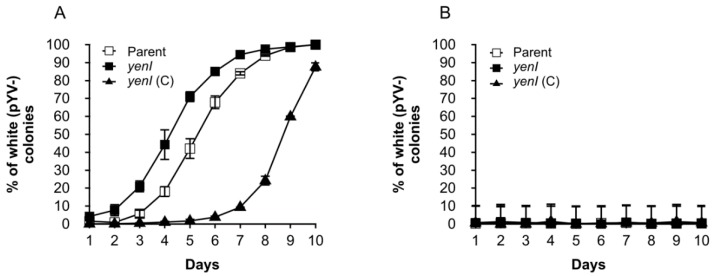
Quorum sensing (QS) impacts on virulence plasmid maintenance. At 37 °C, over 10 days, the number of white, pYVe virulence, plasmid-negative colonies was greater in the *yenI* mutant compared to the parent, and complementation (*yenI* (C)) over-compensated and reduced the rate of plasmid loss to levels well below that of the parent (**A**). At 22 °C, the number of white colonies remained low and stable over ten days with no differences between the parent and QS mutant (**B**).

Since *spyA* expression was reduced in both the *yenI* and *yenI*/*yenR*/*ycoR* mutants but not in the *yenR*/*ycoR* mutant, this suggested that the AHL synthase and by association, the AHLs generated, are involved in the positive control of *spyA*, suggesting that QS is involved in the partition of pYVe during cell division. To investigate this further, the presence of pYVe in the parent and *yenI* population was determined over 10 days by sub-culturing each strain at 37 °C in LB broth, plating serially diluted cultures at 10^−6^ on to CR-MOX agar plates, and assessing the percentage of virulence plasmid-negative, white colonies against red, pYve-positive colonies for each day. Figure 6 reveals that pYVe is lost more rapidly from the *yenI* mutant population when compared with the parent population. For example, after 5 days of serial passage at 37 °C, ~40% of the parent strain had lost pYVe compared with approximately 70% of the *yenI* mutant. Furthermore, when a functional copy of *yenI* was introduced in trans, the mutation was over-compensated, and pYVe was retained for much longer in the population when compared with the parent strain (Figure 6A). However, the exogenous addition of 3-oxo-C6-HSL or C6-HSL to the *yenI* mutant did not restore *spyA* expression to wild type levels (data not shown).

At 22 °C, the stability of pYVe appeared constant in the parent and QS mutants over a 10-day period with the percentage of white, pYVe negative colonies remaining at around 0–1% (Figure 6B).

## 4. Discussion

Members of the *luxR* family of transcriptional regulators are often, but not always, linked to a *luxI* ortholog [36]. For example, in *Pseudomonas aeruginosa*, two *luxR* orthologs, *qscR* and *vqsR*, are unlinked to the *luxI* orthologs, *lasI* and *rhlI*. QscR is an integral component of the *P. aeruginosa* QS system which governs the timing of QS-controlled gene expression. A *qscR* mutant produces the LasI-generated AHL signal, prematurely advancing transcription of a number of QS-regulated genes, while VqsR is responsible for the production of extracellular virulence factors and nematode killing [37]. In *Y. enterocolitica* 8081, *ycoR* is a *luxR* ortholog that is unlinked to an AHL synthase and appears to be the only example of an orphan *luxR* in the *Yersinia* genus. Orphans not only lack a partner *luxI* ortholog, but they are often different in size to other *luxR* homologues, and their translated products may have key conserved amino acid residues missing which are important for DNA- and AHL-binding activities. They may also act independently of any cognate AHL or AHL synthase [36]. Given that YcoR shares considerable homology with other LuxR orthologs in both the *N*- and *C*-terminal domains, this suggests that, functionally, it is likely to possess both DNA- and AHL-binding capabilities.

It is interesting that *ycoR* is present in certain ‘New World’ (North America) strains but absent from Old World (Europe and Japan) strains. Examination of the *ycoR* locus of the *Y. enterocolitica* 8081 strain and the equivalent region in the 90/54 strain (Figure 1) revealed that *ycoR* is absent at this locus in the latter but YE1025, *sfsB*, and YE1027 remain intact. The intergenic region between YE1025 and *ycoR* is conserved but largely missing between YE1027 and *ycoR*. There are some remnant bases within the missing regions, which suggests that the ‘Old World’ strains have lost *ycoR* rather than the USA strains gaining *ycoR.* This loss of genetic material is not uncommon in *Yersinia* with genome rearrangements through sequence insertion and gene deletion being a contributing factor in the abolition and alteration of pre-existing gene expression pathways in the evolution of *Y. pestis* to a hyper-virulent pathogen compared to its closest ancestor, *Y. pseudotuberculosis* [38].

LC-MS of spent culture supernatants of the *Y. enterocolitica* 8081 parent identified a diverse range of AHLs, the most abundant of which were 3-oxo-C6-HSL, C6-HSL and 3-oxo-C7-HSL. AHLs with C7 side chains are uncommon and 3-oxo-C7-HSL is produced by *Y. pseudotuberculosis* [6], while *Rhizobium leguminosarum* [39] and *Serratia marcescens* [40] produce the unsubstituted AHL, C7-HSL. AHL production was abolished in the *yenI* mutants, suggesting that YenI is the only AHL synthase in *Y. enterocolitica* 8081. Furthermore, AHL production was unaffected by deletion of either *yenR*, *ycoR* or both, suggesting that *yenI* is not regulated by either of the LuxR homologues and therefore, in contrast to most QS systems, is not autoinducible. These findings are consistent with our previous observations for the *Y. enterocolitica* strain 10640 where *yenI* was clearly not autoinducible [41].

Although the phenotypes of AHL synthase mutants can usually be restored to wild type by provision of the cognate exogenous AHL, this is not always the case. Production of the purple pigment violacein by *Chromobacterium violaceum* is abolished by mutation of the AHL synthase gene *cviI.* However, violacein production cannot be restored in the *cviI* mutant by exogenous addition of the cognate AHL [42]. Similar observations have been made for both *Y. enterocolitica* [11,41] and *Y. pseudotuberculosis* with respect to, e.g., motility and *spyA* expression. This lack of response to exogenously supplied AHLs is not due to their ability to gain intracellular access. For *C. violaceum* to respond, a second mutation in a repressor gene is required [42] and it is therefore possible that a similar mechanism may operate in *Yersinia*.

In *Y. enterocolitica* 8081, temperature controls motility, and the production of flagella and swimming occurs in the laboratory at temperatures below 30 °C, while at 37 °C, the organism is non-motile [43]. Plate motility assays using two different types of solid media revealed that the 8081 parent and QS mutants are all capable of both swimming and swarming. This contrasts with our previous study on the *Y. enterocolitica* strains 90/54 (serotype O:9) and 10460, where the *yenI* mutation in the former showed impaired swimming motility and was unable to swarm, while there was no observable swimming or swarming motility defect in the 10460 QS mutants [11]. Our observations are also different to those made by Tsai and Winans [12] who found that *Y. enterocolitica* JB580 *yenI* and *yenR* mutants were both motile in plate swimming assays. On swarm plates, however, the *yenI* mutant, in contrast to the JB580 parent, exhibited a hyper-swarming phenotype which could be restored to non-motile parent levels with the addition of 3-oxo-C6-HSL [12]. It is, however, intriguing that 8081 swimming assays in a liquid medium revealed a motility defect in all the QS mutants after 24 h which reflects the plate motility assays conducted for strain 90/54 [11]. This is noteworthy as it is generally accepted that strains with mutations in ‘quorum-hindered’ apo-LuxR homologues, such as YenR described by Tsai and Winans [12] exhibit opposite phenotypes to their *yenI* mutant counterparts. However, our data shows that all of our QS mutants exhibited impaired swimming, such that the *yenI* mutant did not have the opposite swimming phenotype to the *yenR* or *ycoR* single or double mutants. Taken together, these data suggest that the *Y. enterocolitica* QS-associated motility phenotypes are strain dependent with additional layers of complexity within quorum-hindered systems that have yet to be uncovered.

The ability of *Y. enterocolitica* 8081 parent and QS mutants to adhere to human colorectal adenocarcinoma cells (Caco-2) represents a useful model for enteropathogens for which the intestine is the usual site of entry or replication [44] and is highly susceptible to *Yersinia* infection [45]. The attachment of the *yenI* mutant to Caco-2 cells was ~30% lower than the parent and could be complemented with a plasmid-borne copy of *yenI.* More striking was the ~60% reduction in attachment of the *yenR/ycoR* which was also genetically complementable. Since the *yenR and ycoR* single mutants were not impaired in Caco-2 adherence, this suggests that YenR and YcoR may functionally substitute for each other, and it is possible that the YenI AHLs as well as either of the response regulators activate some component of the attachment mechanism of *Y. enterocolitica*.

The qRT-PCR data suggested that *spyA* expression is altered in 8081 carrying a *yenI* mutation. These data are consistent with the plasmid loss assays that suggest that pYVe is lost more readily from QS mutants at 37 °C when compared with the parent strain. An analysis of *spyA* expression revealed that expression in the parent is maximal at the start of the exponential phase of growth (approx. 5.5 h) which would be expected given the metabolic status of the dividing population and the need for pYVe to be partitioned during cell division. The differences in *spyA* expression in the *yenI* and *yenl/yenR/ycoR* mutants compared with the parent strain are significant. However, no changes in *spyA* expression in the *yenR/ycoR* mutant were apparent. This suggests that AHLs associated with YenI may be involved in plasmid maintenance but in association with an as yet unidentified regulator. In this context, *P. aeruginosa*, LuxR ortholog-independent activation of genes by AHLs has been observed [46].

The plasmid loss plate assays revealed that the population is likely to lose pYVe at 37 °C over 10 days compared with a population grown at 22 °C. The biological significance of these observations is unclear as it would seem more logical for a population grown at host temperatures to maintain the plasmid. However, growing the population in conventional rich laboratory media at 37 °C does not closely mimic host conditions, and it is therefore possible that additional triggers are required in vivo to ensure the population maintains the plasmid. In this context, temperature, calcium levels and pH have been shown to influence virulence plasmid stability [47].

Recently Wang et al. [48] showed that during infection of mouse Peyer’s-patches, the copy number of the *Y. pseudotuberculosis* virulence plasmid pYV increased up to 6-fold compared with the infecting inoculum. Our *Y. enterocolitica* data suggest that QS may play a role during infection by regulating pYVe plasmid partition via SpyA. Plasmid copy number and efficient virulence plasmid partition are likely to be central to the maintenance of an efficient *Yersinia* infection. Conceptually, it is logical for the QS system to fulfil this role to ensure that all members of the population maintain and partition pYVe to ensure each individual cell has the propensity to elicit a T3SS response against host cell defences. Further work will be required to fully establish the molecular basis through which QS contributes to virulence plasmid maintenance.

## Supplementary Materials

The following are available online at http://www.mdpi.com/2073-4425/9/6/307/s1, Table S1: Strains used in this study, Table S2: Plasmids used in this study, Table S3: Primers used in this study, Table S4: List of AHLs with percentage of the total, Figure S1: The convergent and overlapping arrangement of *yenR and yenI*, Figure S2: Total AHL production in *Y. enterocolitica* 8081 parent and QS mutants.
