# Supplementary material for: The Quorum Sensing System of Yersinia enterocolitica 8081 Regulates Swimming Motility, Host Cell Attachment, and Virulence Plasmid Maintenance
Source: Genes (Basel). 2018 Jun 20;9(6):307. doi: 10.3390/genes9060307 (PMC6027161; doi:10.3390/genes9060307)
Supplement: Supplementary file 1 [file genes-09-00307-s001.zip › Tables S1-S4.docx]

**Table S1. Strains used in this study**

| **Strain** | **Notes** | | | **Reference/Source** |
| --- | --- | --- | --- | --- |
|  | |  |  |  |
| ***Escherichia coli*** |  | | |  |
| JM109 | *recA1, endA1, gyrA96, thi, hsdR17, supE44, relA1,* Δ*(lac-proAB), mcrA,* [*F′ traD36 proAB lacIq lacZ* Δ*M15*] | | | [1] |
| S17-1 | *thi*-1 *pro hsdR*− *hsdM*+ *recA*::RP4-2-Tcr::Mu; KmR | | | [2] |
| ***Yersinia enterocolitica*** |  | | |  |
| 8081 | Wild-type, serotype O:8, biotype 1B, pYV^+^, sequenced strain | | | [3] |
| 8081 Δ*yenI* | *yenI* chromosomal deletion mutant derived from 8081, Cm^R^ | | | This study |
| 8081 Δ*yenR* | *yenR* chromosomal deletion mutant derived from 8081, Km^R^ | | | This study |
| 8081 Δ*ycoR* | *ycoR* chromosomal deletion mutant derived from 8081, Sm^R^ | | | This study |
| 8081 Δ*yenR*/*ycoR* | *yenR*/*ycoR* chromosomal deletion double mutant derived from 8081, Km^R^, Sm^R^ | | | This study |
| 8081 Δ*yenI*/*yenR*/*ycoR* | *yenI*/*yenR/ycoR* chromosomal deletion triple mutant derived from 8081, Cm^R^, Km^R^, Sm^R^ | | | This study |
| 8081 P_spyA_ Δ*yenI* | *spyA* promotor fused to *luxCDABE* inserted into *Y. enterocolitica* 8081 Δ*yenI* | | | This study |
| 8081 P_spyA_ Δ*yenR*/*ycoR* | *spyA* promotor fused to *luxCDABE* inserted into *Y. enterocolitica* 8081 Δ*yenR*/*ycoR* | | | This study |
| 8081 P_spyA_ Δ*yenI*/*yenR*/*ycoR* | *spyA* promotor fused to *luxCDABE* inserted into *Y. enterocolitica* 8081 Δ*yenR*/*ycoR* Δ*yenI*/*yenR*/*ycoR* | | | This study |

**Table S2. Plasmids used in this study**

| **Plasmid** | **Description** | **Reference/**  **Source** |
| --- | --- | --- |
| pGEMT/easy | Ampicillin resistant PCR product cloning vector | Promega |
| pBluescript SKII+ | Ampicillin resistant cloning vector | Stratagene |
| pDM4 | Chloramphenicol resistant suicide vector | [4] |
| pBlue*lux* | Ampicillin resistant promoter-less *luxCDABE* cassette in a multisite polylinker | [5] |
| pHG327 | Ampicillin resistant low-copy number vector. | [6] |
| pUC4K | Source of kanamycin resistance cassette | Pharmacia |
| pACYC184 | Source of chloramphenicol resistance cassette | [7] |
| pHP45Ω | Source of streptomycin resistance cassette | [8] |
| pME3087 | Tetracycline resistant low-copy number vector. | [9] |
| pAJD434 | λ red recombinase vector | [10] |
| pYK801 | pDM4 containing *P_spyA_::luxCDABE* | This study |
| pME::*yenI* | pME3087 carrying *yenI* for complementation | This study |
| pME::*yenRycoR* | pME3087 carrying *yenR* and *ycoR* for complementation | This study |

**Table S3. Primers used in this study**

| **Name** | **Primer sequence (5’-3’)** |
| --- | --- |
|  | **Amplification of QS genes** |
| *yenR*f | gtgaggatatgttatacc |
| *yenR*r | gagagtacatcaggttg |
| *yenI*f | ctgcactcgctaagtctc |
| *yenI*r | ccaagcacgcaataagg |
| *ycoR*f | ggattttattaaggaggtg |
| *ycoR*r | ccaagtaagggagcatag |
|  |  |
|  | **Primers for mutagenesis** |
| *yenR*Kan-f | aattgtattgttacattatacacagagtagaattggcctattatgataattgaaagccacgttgtgtctcaa |
| *yenR*Kan-r | aagtttcaactctatgccaagccttattgcgtgcttggcatttaaaacaccttagaaaaactcatcgagcat |
| *yenI*Cm-f | cgtgtacgatgttgttttaattaaataactttggtttttattatgttaaaagttgatcggcacgtaagaggt |
| *yenI*Cm-r | agcacgcaataaggcttggcatagagttgaaacttattaaacctatttaatttacgccccgccctgccactc |
| *ycoR*Sm-f | ctcgaaaaatacagaaaaatcagatatgcatatgcaataatgaataagagagttttcatggcttgttatgac |
| *ycoR*Sm-r | ggatggatcaagaaaacacttggccattatctttgtatactaggaataaacttatttgccgactaccttggt |
|  |  |
|  | **Primers to amplify the *ycoR* locus** |
| *ycoR*flankF | cacaatctcactcaaggc |
| *ycoR*flankR | gcgtatccagatccatc |
|  |  |
|  | **Primers for QRT-PCR** |
| *dnaE*-f | ccaccggacaggtcagctt |
| *dnaE*-r | aactcacgggcggtcattt |
| *invA*-f | cggtgaccacagggcttatt |
| *invA*-R | tgatcgacccccagtgtaatg |
| *yadA*-f | cattgcggttggtgctagtg |
| *yadA*-r | agcgcccacagcaactg |
| *spyA*-f | gatgcgactgatcctcaagct |
| *spyA*-r | tgcaggtcgggaacatagc |

**Table S4. List of AHLs with percentage of the total**

| **AHLs** | **Percentage** |
| --- | --- |
| C4-HSL | 0.6164 |
| C6-HSL | 27.4266 |
| C8-HSL | 0.2626 |
| C12-HSL | 0.0337 |
| C14-HSL | 0.2009 |
| 3-oxo-C6-HSL | 62.4219 |
| 3-oxo-C8-HSL | 1.2484 |
| 3-oxo-C7-HSL | 5.1498 |
| 3-oxo-C10-HSL | 0.0071 |
| 3-oxo-C12-HSL | 0.0371 |
| 3-oxo-C14-HSL | 0.0808 |
| 3-OH-C4-HSL | 0.7140 |
| 3-OH-C6-HSL | 1.7127 |
| 3-OH-C8-HSL | 0.0417 |
| 3-OH-C12-HSL | 0.0275 |
| 3-OH-C14-HSL | 0.0188 |

*N*-butanoylhomoserine lactone (C4-HSL), C8-HSL, *N*-dodecanoylhomoserine (C12-HSL), *N*-tetradecanoylhomoserine lactone (C14-HSL), *N*-(3-oxoheptanoyl)homoserine lactone (3-oxo-C7-HSL), 3-oxo-C8-HSL, *N*-(3-hydroxybutanoyl)homoserine lactone (3-OH-C4-HSL), *N*-(3-hydroxyhexanoyl)homoserine lactone (3-OH-C6-HSL), *N*-(3-hydroxyoctanoyl)homoserine lactone (3-OH-C8-HSL), *N*-(3-hydroxydecanoyl)homoserine lactone (3-OH-C12-HSL), and *N*-(3-hydroxytetradecanoyl)homoserine lactone (3-OH-C14-HSL)).

1. Yanischperron, C.; Vieira, J.; Messing, J. Improved M13 phage cloning vectors and host strains-nucleotide-sequences of the m13mp18 and puc19 vectors. *Gene* **1985**, *33*, 103–119.

2. Simon, R.; Priefer, U.; Puhler, A. a broad host range mobilization system for *in vivo* genetic engineering: transposon mutagenesis in gram negative bacteria. *Nat.Biotechnol.* **1983**, *1*, 784–791.

3. Portnoy, D. A.; Moseley, S. L.; Falkow, S. Characterization of plasmids and plasmid-associated determinants of *Yersinia enterocolitica* pathogenesis. *Infect.Immun.* **1981**, *31*, 775–782.

4. O'Toole, R.; Milton, D. L.; Wolf-Watz, H. Chemotactic motility is required for invasion of the host by the fish pathogen *Vibrio anguillarum*. *Mol.Microbiol.* **1996**, *19*, 625–637.

5. Atkinson, S.; Chang, C. Y.; Patrick, H. L.; Buckley, C. M. F.; Wang, Y.; Sockett, R. E.; camara, M.; williams, P. Functional interplay between the *Yersinia pseudotuberculosis* YpsRI and YtbRI quorum sensing systems modulates swimming motility by controlling expression of *flhDC* and *fliA*. *Mol.Microbiol.* **2008**, *69*, 137–151.

6. Stewart, G. S. A. B.; Lubinskymink, S.; Jackson, C. G.; Cassel, A.; Kuhn, J. pHG165 - a pBR322 copy number derivative of pUC8 for cloning and expression. *Plasmid* **1986**, *15*, 172–181.

7. CHANG, A.; Cohen, S. N. Construction and characterization of amplifiable multi-copy DNA cloning vehicles derived from p15a cryptic mini-plasmid. *J.Bacteriol.* **1978**, *134*, 1141–1156.

8. Prentki, P.; Krisch, H. M. *In vitro* insertional mutagenesis with a selectable DNA fragment. *Gene* **1984**, *29*, 303–313.

9. voisard, C.; RELLA, M.; Haas, D. Conjugative transfer of plasmid rp1 to soil isolates of *Pseudomonas fluorescens* is facilitated by certain large rp1 deletions. *FEMS Microbiol.Lett.* **1988**, *55*, 9–13.

10. Maxson, M. E.; Darwin, A. J. Identification of inducers of the *Yersinia enterocolitica* phage shock protein system and comparison to the regulation of the RpoE and Cpx extracytoplasmic stress responses. *J.Bacteriol.* **2004**, *186*, 4199–4208.
