# Supplementary material for: The Quorum Sensing System of Yersinia enterocolitica 8081 Regulates Swimming Motility, Host Cell Attachment, and Virulence Plasmid Maintenance
Source: Genes (Basel). 2018 Jun 20;9(6):307. doi: 10.3390/genes9060307 (PMC6027161; doi:10.3390/genes9060307)
Supplement: Supplementary file 1 [file genes-09-00307-s001.zip › Supplementary Fig S2 v3.pptx]

## Slide 1
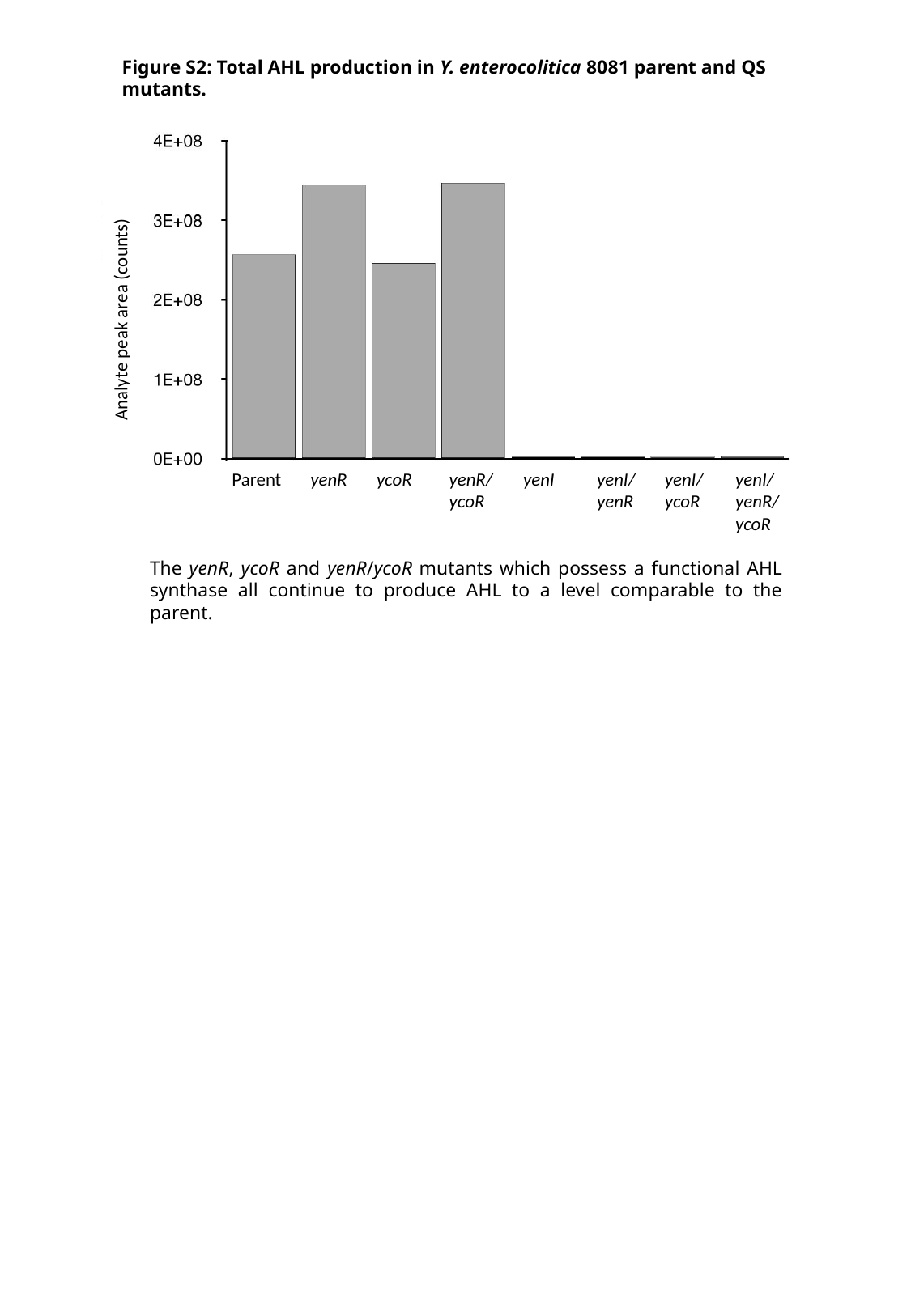

Figure S2: Total AHL production in Y. enterocolitica 8081 parent and QS mutants.
Analyte peak area (counts)
Parent
yenR
yenI
ycoR
yenI/ ycoR
yenI/
yenR/ ycoR
yenR/ ycoR
yenI/ yenR
The yenR, ycoR and yenR/ycoR mutants which possess a functional AHL synthase all continue to produce AHL to a level comparable to the parent.
